# Material Extrusion 3D Printing (ME3DP) Process Simulations of Polymeric Porous Scaffolds for Bone Tissue Engineering

**DOI:** 10.3390/ma16062475

**Published:** 2023-03-20

**Authors:** Ramsha Imran, Ans Al Rashid, Muammer Koç

**Affiliations:** 1 Division of Sustainable Development, College of Science and Engineering, Hamad Bin Khalifa University, Qatar Foundation, Doha 34110, Qatar; 2 Faculty of Engineering, University of Karabük, Karabük 78050, Turkey

**Keywords:** 3D printing, bone tissue engineering, process simulation, porous scaffolds, biodegradable

## Abstract

Bone tissue engineering (BTE) is an active area of research for bone defect treatment. Some polymeric materials have recently gained adequate attention as potential materials for BTE applications, as they are biocompatible, biodegradable, inexpensive, lightweight, easy to process, and recyclable. Polyetherimide (PEI), acrylonitrile butadiene styrene (ABS), and polyamide-12 (PA12) are potential biocompatible materials for biomedical applications due to their excellent physical, chemical, and mechanical properties. The current study presents preliminary findings on the process simulations for 3D-printed polymeric porous scaffolds for a material extrusion 3D printing (ME3DP) process to observe the manufacturing constraints and scaffold quality with respect to designed structures (porous scaffolds). Different unit cell designs (ventils, grid, and octet) for porous scaffolds, virtually fabricated using three polymeric materials (PEI, ABS, and PA12), were investigated for process-induced defections and residual stresses. The numerical simulation results concluded that higher dimensional accuracy and control were achieved for grid unit cell scaffolds manufactured using PEI material; however, minimum residual stresses were achieved for grid unit cell scaffolds fabricated using PA12 material. Future studies will include the experimental validation of numerical simulation results and the biomechanical performance of 3D-printed polymeric scaffolds.

## 1. Introduction

Bone tissue engineering (BTE) is an active area of research for bone defect treatment [1,2,3,4], in which bio-scaffolds are implanted to heal the bone damage caused by accidents, infections, or tumors [5]. The scaffold is essential in providing a template for cell adhesion, cell proliferation, and structural support to newly formed bone [6]. Metals, alloys, polymers, ceramics, and composites are commonly used for such applications [7,8,9]. However, some limitations prevail in the widespread use of each class of materials, such as mismatch of mechanical properties for metals, insufficient mechanical properties of polymers, the release of metallic ions in the case of alloys, and brittleness in ceramics [10,11,12,13].

The search for biomaterials in BTE applications is ongoing to achieve the desired physical, mechanical, and biological properties of scaffolds. Some polymeric materials have recently gained adequate attention as potential materials for biomedical applications, as they are biocompatible, biodegradable, inexpensive, lightweight, easy to process, and recyclable [14]. Polylactic acid (PLA) [15,16], poly (lactic-co-glycolic acid) (PLGA) [17], polycaprolactone (PCL) [18], and polyether ether ketone (PEEK) [19] are FDA-approved biomaterials [20]. These materials possess significant mechanical properties comparable to the natural bone; therefore, they can act as load-bearing prosthetic elements [21]. Polyetherimide (PEI) is another biocompatible material under investigation for biomedical applications due to its excellent physical, chemical, and mechanical properties [22]. In addition, PEI is observed to have stiffness and physiological structure in charge transfer comparable to natural bone [23].

The scaffold is expected to allow for the flow of body fluids through interconnected porous structures and provide a template for cell growth [24,25]. However, the fabrication of such complex structures is crucial, and the manufacturing process should provide high reproducibility and accuracy [26]. Metal powder sintering [27], polymeric sponge replication [28], investment casting [29], and gas foaming [30] are some conventional manufacturing processes used for the fabrication of 3D scaffolds. The research on the manufacturing of porous scaffolds is mainly at the lab scale; however, the recent innovations in biomaterials for such applications have opened up the opportunity to adopt advanced manufacturing processes as an alternative to producing on-demand porous scaffolds for BTE precisely and accurately [31]. Therefore, advanced manufacturing techniques, such as additive manufacturing (AM) or 3D printing (3DP), are rapidly growing in several sectors [32,33], including biomedical research [34,35,36]. These processes provide higher freedom to design and rapidly fabricate complex structures (such as 3D porous scaffolds) with higher precision and accuracy and lower material wastage [37].

Several studies reported using AM processes to fabricate porous scaffolds for BTE. For instance, Tang et al. [38] manufactured PEI scaffolds through a 3DP process and evaluated their physical, mechanical, and biological performance. Likewise, Suffo and Revenga [39] investigated the biomechanical performance of a commercial polymer (ULTEMTM 1010) for knee replacement. Polyakov et al. [40] performed thermomechanical analysis on 3D-printed carbon nanofiber-reinforced PEI nanocomposites. In addition, acrylonitrile butadiene styrene (ABS) and polyamide (PA) materials have been investigated for biomedical applications due to their excellent mechanical properties [41]. Alblooshi [42] reported the fabrication of chemically functionalized polymeric (PLA, ABS, and polyethylene terephthalate (PET)) porous scaffolds using the 3DP process. Alkebsi et al. [43] recently reported a novel design for 3D-printed variable porosities of porous scaffolds using ABS material. The mechanical properties of designed scaffolds were evaluated using the finite element method (FEM), numerical model, and experimental testing. Likewise, Li et al. [44] manufactured polyamide-12 (PA12)/hydroxyapatite (HA) porous scaffolds with gradient structures using the selective laser sintering (SLS) process. The effect of porosity and pore type on mechanical properties was analyzed using experimental and FEM simulations. Zhao et al. [45] designed triply periodic minimal surface (TPMS)-based porous scaffolds fabricated using pure PA12 and PA12/HA materials via the SLS process. Mechanical and hydrophilicity tests were conducted to observe the effect of HA addition to the PA12 polymer.

The literature concludes that polymers and their composites can provide low-cost and reliable BTE scaffolds with significant mechanical properties. Most of the reported literature mainly utilized experimental techniques for AM of biomaterials; however, numerical modeling and simulation tools can provide an easier, more straightforward, and inexpensive performance evaluation of 3D-printed structures [46]. The dimensional control and precision of 3D-printed porous scaffolds for BTE are vital; therefore, it is essential to evaluate the thermomechanical performance of different materials, designs, and process parameters to achieve the desired dimensions of the final product [47]. The numerical simulations can estimate process-induced defects and residual stresses that can be addressed before fabrication to save resources and costs associated with experimental investigations. Al Rashid and Koç [48,49,50,51] reported experimental validations on deformations, distortions, and mechanical properties for different materials, product designs, and process parameters using Digimat software (version 2021.3, from e-Xstream engineering, Käerjeng, Luxembourg). The predictability of Digimat software was found to be in good agreement with the experimental results.

Given the existing literature in the field of additively manufactured polymer scaffolds, there is a pressing need to continue the pursuit of potential biomaterials and explore the manufacturability of complex polymeric scaffold structures. Therefore, in this study, process simulations for 3D-printed polymeric scaffolds were performed for the material extrusion 3D printing (ME3DP) process to observe the manufacturing constraints and scaffold quality with respect to designed geometries. Different scaffold designs (ventils, grid, and octet) virtually fabricated with polymeric materials (PEI, ABS, and PA12) were investigated for process-induced defections and residual stresses.

## 2. Materials and Methods

The main aim of this study is to analyze the effect of different polymeric materials and the design of unit cells on dimensional control, accuracy, thermal variations, and residual stresses of additively manufactured porous scaffolds. The subsequent sections report details on the design of porous scaffolds, methodology, and ME3DP process simulation setup.

### 2.1. Design of Porous Scaffolds

**Figure 1:**
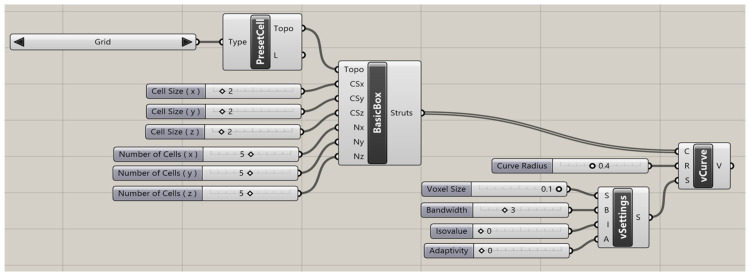
Design of Scaffolds Workflow in Grasshopper.

**Figure 2:**
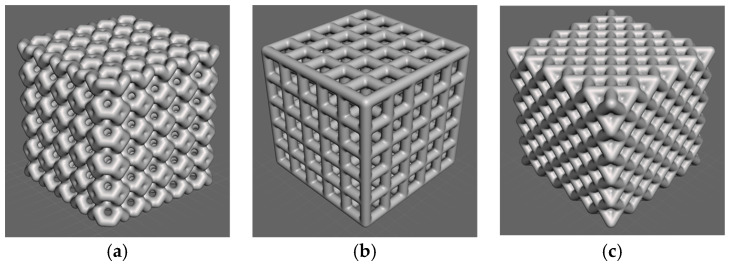
Designed scaffolds using Grasshopper, (**a**) ventils, (**b**) grid, and (**c**) octet.

The porous scaffolds with variable porosity were designed using a Grasshopper^®^ plug-in within Rhino 7^®^ software (from Robert McNeel & Associates, Seattle, WA, USA). Intralattice, a plug-in for Grasshopper^®^ [52], provided an easier and more straightforward workflow for scaffold design, as shown in Figure 1. Three unit cell designs (ventils, grid, and octet) were selected for investigation. A unit cell size of 2 mm was chosen for all the unit cell designs, and five unit cells were produced in each principal direction (i.e., X-, Y-, and Z-axes) to achieve a cubic porous scaffold with overall dimensions of 10 mm × 10 mm × 10 mm. The porosity for different unit cell designs was evaluated using Equation (1), where $V_{Void}$ refers to the void volume and $V_{Total}$ refers to the total bound volume of a solid cube measuring 10 mm × 10 mm × 10 mm. The designed scaffolds exhibited variable porosity levels of 48%, 67%, and 13% for ventils, grid, and octet scaffolds, respectively. Three different scaffolds designed using Grasshopper^®^ are reported in Figure 2.

(1) $$\emptyset =\frac{V_{Void}}{V_{Total}}$$

### 2.2. Methodology

Three different unit cell designs (ventils, grid, and octet) for porous scaffolds and three polymeric materials (PEI, ABS, and PA12) led to a combination of nine numerical simulations, as reported in Table 1. Numerical simulations were performed for each material and each unit cell design. The designed scaffolds in STL format were imported to slicing software (Cura^®^ version 5.0.0, from Ultimaker, Netherlands) to define the toolpath and ME3DP process parameters. The material extrusion temperatures, build plate temperatures, and printing speeds were selected based on experience and reported literature (Table 1). A layer height of 0.1 mm and 100% infill density with a concentric infill pattern were selected, and g-codes were generated for each case to be used in process simulations.

### 2.3. ME3DP Process Simulation

**Figure 3:**
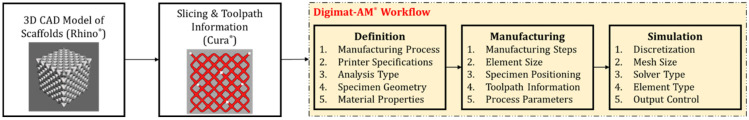
Overall workflow for ME3DP process simulations.

The thermomechanical numerical simulations for the ME3DP process were performed within Digimat software [53], providing flexibility to simulate the ME3DP process for different materials, geometries, and process parameters. The overall workflow for the ME3DP process simulations is presented in Figure 3.

**Figure 4:**
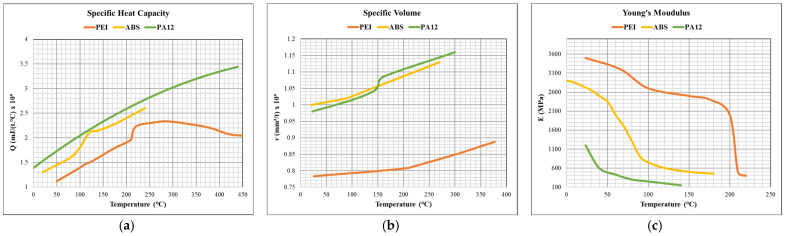
Thermomechanical properties of the polymeric materials used for ME3DP process simulations: (**a**) specific heat capacity, (**b**) specific volume, and (**c**) Young’s modulus.

First, the 3D model of the designed scaffold was imported into the Digimat-AM module of the Digimat^®^ software. The ME3DP printer specifications were identified in the first step, as per Ultimaker 3 extended^®^ (from Ultimaker, Netherlands, with a bed size of 215 mm × 215 mm × 300 mm and a moving platform). The thermomechanical numerical model was selected to better approximate process-induced defects based on the thermal variations during and after the ME3DP process. The polymeric material properties were assigned to the imported scaffold from the Digimat-MX^®^ database [53]. The isotropic material properties were used for all the materials, while temperature-dependent specific heat capacity, specific volume, and young’s modulus were used, as reported in Figure 4. Generally, all the materials were observed to have increased specific heat capacity and specific volume with an increase in temperature; however, young’s modulus decreased at elevated temperatures. The thermal conductivities were 0.22 mW/(mm.°C) for PEI, 0.18 mW/(mm.°C) for ABS, and 0.30 mW/(mm.°C) for PA12 material.

In the subsequent step, the g-code file was imported to define the toolpath for ME3DP simulation, and manufacturing parameters were identified. In addition to extrusion and build plate temperatures, other parameters included chamber temperature (25 °C), bead width (0.4 mm), and convection coefficient (0.015 mW/(mm^2^.°C). A maximum element and voxel size of 0.1 mm was chosen to comply with the layer height selected for the g-code generation. Finally, a layer-by-layer discretization technique was adopted to reduce computation time and costs [48]. Once the job was completed, the temperature, displacement, and residual stress fields were analyzed, and the same procedure was adopted for each case.

## 3. Results and Discussions

The displacement and von Mises stress fields were obtained after successfully completing the numerical simulation. The maximum displacements and von Mises (residual) stresses for different scaffold designs and polymeric materials obtained from numerical simulation results are reported in Table 2. The displacement and residual stress fields exported from Digimat^®^ are presented and discussed in the subsequent sections.

### 3.1. Displacement Fields

**Figure 5:**
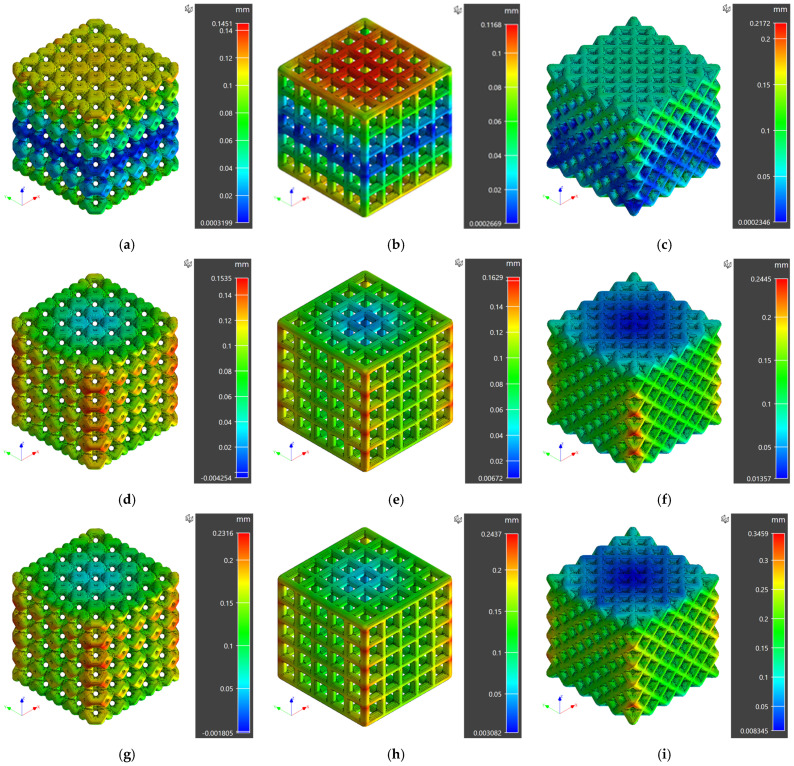
Displacement fields for porous scaffolds for all the cases of designed numerical simulation study (**a**) PEI Porous Scaffold with Ventils Unit Cells (**b**) PEI Porous Scaffold with Grid Unit Cells (**c**) PEI Porous Scaffold with Octet Unit Cells (**d**) ABS Porous Scaffold with Ventils Unit Cells (**e**) ABS Porous Scaffold with Grid Unit Cells (**f**) ABS Porous Scaffold with Octet Unit Cells (**g**) PA12 Porous Scaffold with Ventils Unit Cells (**h**) PA12 Porous Scaffold with Grid Unit Cells (**i**) PA12 Porous Scaffold with Octet Unit Cells.

The displacement fields for different investigated scaffold designs are presented in Figure 5. A maximum displacement of 0.1451 mm, 0.1168 mm, and 0.2172 mm was observed for ventils, grid, and octet unit cell scaffolds in the case of PEI material. For ABS material, a maximum displacement of 0.1535 mm, 0.1629 mm, and 0.2445 mm was recorded for ventils, grid, and octet unit cell scaffolds, respectively. Finally, a maximum displacement of 0.2316 mm, 0.2437 mm, and 0.3459 mm was achieved for ventils, grid, and octet unit cell scaffolds for PA12 material.

It is evident from displacement fields that the minimum variation from designed scaffold structures (lower displacements) was observed for PEI material. For instance, for ventils unit cell scaffolds, a maximum displacement of 0.1451 mm was recorded for PEI material, following higher displacements of 0.1535 mm and 0.2316 mm for ABS and PA12 material, respectively. A similar trend was observed for grid and octet unit cell scaffolds.

Higher maximum displacements for ABS and PA12 materials are attributed to their higher specific heat capacity and specific volume than PEI material. These two material properties are vital in dimensional control as the extrudate goes through several heat transfer phenomena during the extrusion, deposition, and solidification processes. From numerical simulation results, it is concluded that higher dimensional accuracy and control were achieved for grid unit cell scaffolds manufactured using PEI material. Although the observed deviations from the designed parts are relatively lower, in the case of BTE applications, it is essential to produce the net shape per patient-specific requirements [42].

### 3.2. Residual Stress Fields

**Figure 6:**
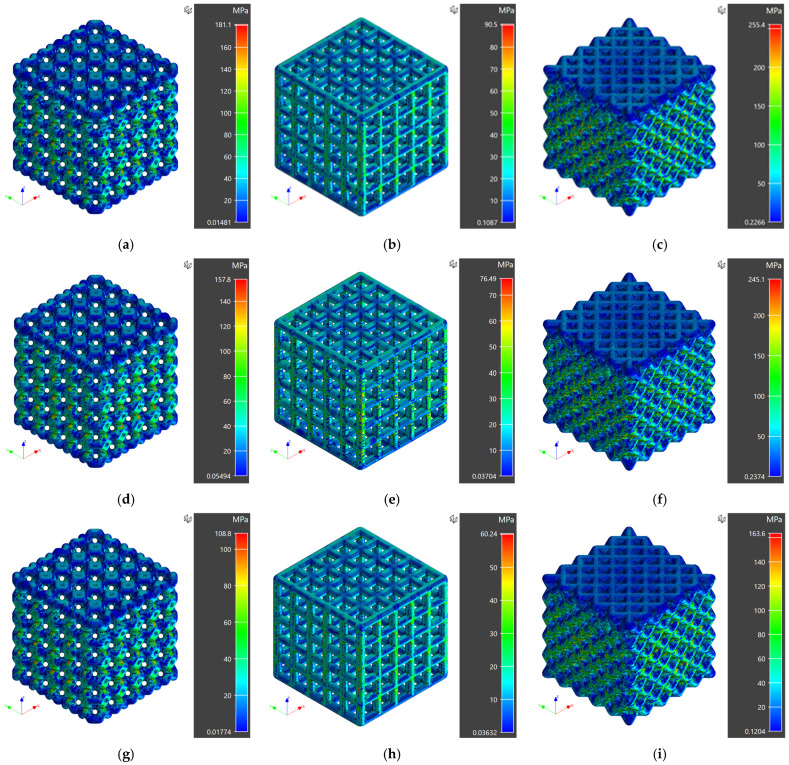
Residual stresses for porous scaffolds for all the cases of designed numerical simulation study (**a**) PEI Porous Scaffold with Ventils Unit Cells (**b**) PEI Porous Scaffold with Grid Unit Cells (**c**) PEI Porous Scaffold with Octet Unit Cells (**d**) ABS Porous Scaffold with Ventils Unit Cells (**e**) ABS Porous Scaffold with Grid Unit Cells (**f**) ABS Porous Scaffold with Octet Unit Cells (**g**) PA12 Porous Scaffold with Ventils Unit Cells (**h**) PA12 Porous Scaffold with Grid Unit Cells (**i**) PA12 Porous Scaffold with Octet Unit Cells.

The residual stress fields for different investigated scaffold designs are presented in Figure 6. A maximum von Mises stress of 181.1 MPa, 90.5 MPa, and 255.4 MPa was observed for ventils, grid, and octet unit cell scaffolds in the case of PEI material. For ABS material, a maximum von Mises stress of 157.8 MPa, 76.49 MPa, and 245.1 MPa was recorded for ventils, grid, and octet unit cell scaffolds, respectively. Finally, a maximum von Mises stress of 108.8 MPa, 60.24 MPa, and 163.6 MPa was achieved for ventils, grid, and octet unit cell scaffolds for PA12 material.

It is evident from residual stress fields that the minimum residual stresses for scaffold structures were observed for grid unit cell design fabricated using PA12 material. For instance, for PA12 grid unit cell scaffolds, maximum residual stress of 60.24 MPa was recorded, followed by residual stresses of 108.8 MPa and 163.6 MPa for ventils and octet unit cell scaffolds, respectively. A similar trend was observed for PEI and ABS materials. Higher residual stresses for PEI are attributed to a higher young’s modulus than ABS and PA12 materials. Higher residual stresses were observed for PEI for all the scaffold designs, followed by ABS and PA12, consistent with the temperature-dependent material properties. From numerical simulation results, it is concluded that minimum residual stresses were achieved for grid unit cell scaffolds manufactured using PA12 material. The residual stresses can play an essential role during the application phase of the designed structures [54]; therefore, it is vital to predict and minimize these stresses for proper functioning at the host site.

## 4. Conclusions

There is a pressing need to continue the pursuit of potential biomaterials and explore the manufacturability of complex PEI scaffold structures. Therefore, in this study, the process simulations for 3D-printed polymeric scaffolds were performed for the material extrusion 3D printing (ME3DP) process to observe the manufacturing constraints and scaffold quality with respect to designed parts. Different scaffold designs and polymeric materials were investigated for process-induced defections and residual stresses. The numerical simulation model provides a cheaper solution to multi-dimensional optimization linked with the ME3DP process (i.e., material properties, product design, and process parameters). The numerical simulation results expressed that higher dimensional accuracy and control were achieved for grid unit cell scaffolds manufactured using PEI material; however, minimum residual stresses were achieved for grid unit cell scaffolds fabricated using PA12 material. The numerical simulation results will be validated with experimental observations for future studies. The current analysis was performed using different materials and scaffold designs while process parameters were kept constant; they will be varied, and the effect on dimensional control and 3D-printed product quality will be investigated. Furthermore, the numerical modeling approach can be adopted before fabrication to complement sustainability in terms of materials and resource utilization. The proposed methodology and numerical model can be adopted widely for biomedical applications where patient-specific implants are desired and manufactured on demand.

## Figures and Tables

**Table 1:** Numerical simulation study design and ME3DP process parameters.

No.	Material	Unit Cell Design	Extrusion Temperature	Build Plate Temperature	Printing Speed (mm/s)
1	PEI	Ventils	360 °C	100 °C	70
2		Grid			
3		Octet			
4	ABS	Ventils	230 °C	80 °C	55
5		Grid			
6		Octet			
7	PA12	Ventils	245 °C	60 °C	70
8		Grid			
9		Octet			

**Table 2:** A summary of maximum displacement and residual stresses for porous scaffolds.

No.	Material	Unit Cell Design	Maximum Displacement (mm)	Von Mises Stress (MPa)
1	PEI	Ventils	0.1451	181.1
2		Grid	0.1168	90.5
3		Octet	0.2172	255.4
4	ABS	Ventils	0.1535	157.8
5		Grid	0.1629	76.49
6		Octet	0.2445	245.1
7	PA12	Ventils	0.2316	108.8
8		Grid	0.2437	60.24
9		Octet	0.3459	163.6

## Data Availability

Data will be made available on reasonable request to the corresponding author.
